# Evaluation of Neurotoxicity With Human Pluripotent Stem Cell–Derived Cerebral Organoids

**DOI:** 10.1002/cpz1.744

**Published:** 2023-04-17

**Authors:** Thomas Parmentier, Jonathan LaMarre, Jasmin Lalonde

**Affiliations:** ^1^ Department of Biomedical Sciences, Ontario Veterinary College University of Guelph Guelph Ontario Canada; ^2^ Department of Molecular and Cellular Biology, College of Biological Science University of Guelph Guelph Ontario Canada; ^3^ Current address: Département de Sciences Cliniques, Faculté de Médecine Vétérinaire Université de Montréal Saint‐Hyacinthe Québec Canada

**Keywords:** calcium imaging, cerebral organoids, microelectrode arrays, neurogenesis, neurotoxicity

## Abstract

The recent development of human cerebral organoids provides an invaluable *in vitro* model of human brain development to assess the toxicity of natural or man‐made toxic substances. By recapitulating key aspects of early human neurodevelopment, investigators can evaluate with this three‐dimensional (3D) model the effect of certain compounds on the formation of neuronal networks and their electrophysiological properties with more physiological relevance than neurons grown in monolayers and in cultures composed of a unique cell type. This promising potential has contributed to the development of a large number of diverse protocols to generate human cerebral organoids, making interlaboratory comparisons of results difficult. Based on a previously published protocol to generate human cortical organoids (herein called cerebral organoids), we detail several approaches to evaluate the effect of chemicals on neurogenesis, apoptosis, and neuronal function when exogenously applied to cultured specimens. Here, we take as an example 4‐aminopyridine, a potassium channel blocker that modulates the activity of neurons and neurogenesis, and describe a simple and cost‐effective way to test the impact of this agent on cerebral organoids derived from human induced pluripotent stem cells. We also provide tested protocols to evaluate neurogenesis in cerebral organoids with ethynyl deoxyuridine labeling and neuronal activity with live calcium imaging and microelectrode arrays. Together, these protocols should facilitate the implementation of cerebral organoid technologies in laboratories wishing to evaluate the effects of specific compounds or conditions on the development and function of human neurons with only basic cell culture equipment. © 2023 The Authors. Current Protocols published by Wiley Periodicals LLC.

**Basic Protocol 1**: Generation of human cerebral organoids from pluripotent stem cells

**Support Protocol 1**: Human pluripotent stem cell culture

**Basic Protocol 2**: Evaluation of neurogenesis in cerebral organoids with ethynyl deoxyuridine labeling

**Basic Protocol 3**: Calcium imaging in cerebral organoids

**Basic Protocol 4**: Electrophysiological evaluation of cerebral organoids with microelectrode arrays

**Support Protocol 2**: Immunostaining of cerebral organoids

## INTRODUCTION

Evaluation of the neurotoxicity of compounds in humans is usually performed through a variety of *in vitro* assays, such as incubating *in vitro*–cultured neurons with the compound, or *in vivo* evaluation by administering it to laboratory animals. Although the use of these complementary models decreases the risk for unforeseen neurotoxicity, evaluating neurotoxicity in the developing human brain remains challenging. Indeed, this stage of development has traditionally been inaccessible for evaluation due to technical and ethical considerations, highlighting the need for human‐specific complex models of brain development. Cerebral organoids are three‐dimensional (3D) cellular aggregates of neural cells grown in a specific manner to recapitulate key aspects of brain development, including layered organization, diversity of neural cell types, and complex pattern of spontaneous activity. The development of cerebral organoids from human induced pluripotent stem cells (iPSCs) provides unprecedented access to developing neural cells at the cellular and molecular resolution and fills an important gap in existing tools to test the neurotoxicity of compounds. By testing the effect of compounds directly on human iPSC‐derived cerebral organoids, investigators are now able to assess the potential neurotoxicity of these compounds on brain development and neuronal function using a variety of phenotypic assays available for cerebral organoids. In this article, we provide detailed instructions on how to set up an efficient and cost‐effective culture system for generating cerebral organoids from human iPSCs. We also share essential protocols to assess neurogenesis within the organoids and neuronal activity at the single‐cell and neuronal‐network level. These different protocols should allow researchers with limited experience in cell culture to implement this relevant model of human brain development in their laboratories.

**Figure 1:**
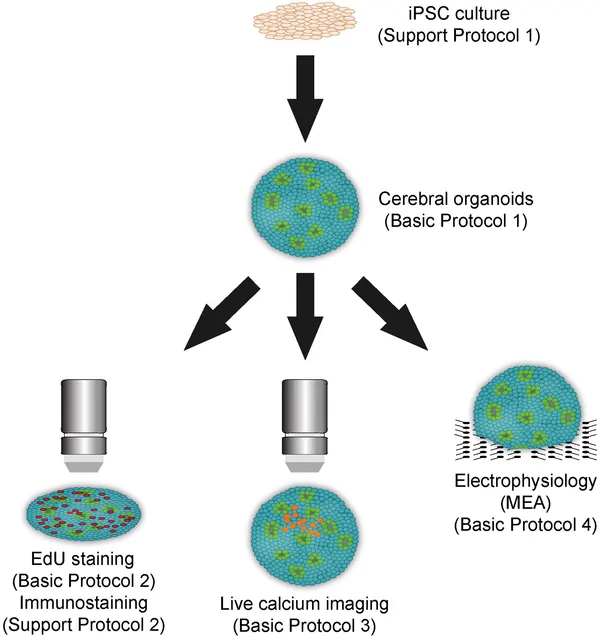
Schematic workflow of the different protocols. EdU, ethynyl deoxyuridine; iPSC, induced pluripotent stem cell; MEA, microelectrode array.

In Basic Protocol 1, we present a simple and scalable method to generate human cerebral organoids from iPSCs. In Basic Protocol 2, we describe the evaluation of neuronal differentiation in cerebral organoids using ethynyl deoxyuridine (EdU) labeling. Basic Protocol 3 details the use of live calcium imaging to study neuronal activity at the single‐cell level, and Basic Protocol 4 features electrophysiological analysis of cerebral organoids with microelectrode arrays (MEAs) to investigate neuronal function at the network level (Fig. 1). Support Protocol 1 details the culture of human iPSCs that are used as the primary component of cerebral organoids, and Support Protocol 2 explains the different steps to perform fluorescent immunostaining in cerebral organoids.

## STRATEGIC PLANNING

Before beginning, the user should have actively growing PSCs (e.g., iPSCs). These cells can be purchased directly from vendors (e.g., ATCC) or generated by reprogramming somatic cells. Reprogramming methods are beyond the scope of this article, but detailed protocols have been published about this topic (see *Current Protocols* articles: Rajarajan et al., 2012; Wiley et al., 2017). Cerebral organoids can also be generated from established human embryonic stem cell (ESC) lines. Ethics committee approval is required for the use of ESCs and iPSCs. Support Protocol 1 details the culture of PSCs before cerebral organoid generation.

*CAUTION*: Human iPSCs are a Biosafety Level 2 (BSL‐2) hazard. Follow all appropriate guidelines and regulations for the use and handling of biological hazards.

*NOTE*: All procedures using human iPSCs must first be reviewed and approved by an institutional review board or must follow local guidelines for the use of human samples.

*NOTE*: All procedures pertaining to the culture of stem cells or cerebral organoids must be performed using sterile materials and aseptic technique.

## Basic Protocol 1 GENERATION OF HUMAN CEREBRAL ORGANOIDS FROM PLURIPOTENT STEM CELLS

In this protocol, we describe an easy and cost‐effective approach to generate cerebral organoids using a guided protocol modified from Yoon et al. (2019). Cerebral organoids are obtained through sequential use of small molecules (SMAD inhibitors) and growth factors to obtain cell types normally present in the dorsal forebrain. This guided approach reduces variability in cell composition between organoids, which can be observed with unguided protocols, and increases consistency in subsequent assays using the organoids. This protocol starts from human iPSCs in culture and generates embryoid bodies that are progressively differentiated into cerebral organoids. With longer times in culture, neurons in cerebral organoids progressively acquire a more mature neuronal phenotype with robust electrophysiological properties. Notably, glial cells (astrocytes) also progressively appear in cerebral organoids. This protocol is fully scalable to obtain the desired number of cerebral organoids for neurotoxicity assays.

### Materials

Human iPSCs (e.g., ATCC, cat. no. BXS‐0117; see Support Protocol 1) in a 6‐well plate or 10‐cm dish
Dulbecco's phosphate‐buffered saline (DPBS), without calcium or magnesium (e.g., Thermo Fisher Scientific, cat. no. 14190144)
Accutase (e.g., Stemcell Technologies, cat. no. 07920)
E8 medium (e.g., Stemcell Technologies, cat. no. 05990)
Rho‐associated protein kinase (ROCK) inhibitor Y27632 (e.g., Selleckchem, cat. no. S1049)
E6 medium (e.g., Stemcell Technologies, cat. no. 05946)
Dorsomorphin (see recipe)
SB431542 (see recipe)
Neural medium (see recipe)
Basic fibroblast growth factor (bFGF), recombinant human (see recipe)
Epidermal growth factor (EGF), recombinant human (see recipe)
Brain‐derived neurotrophic factor (BDNF), recombinant human (see recipe)
Neurotrophin‐3, recombinant human (see recipe)

Cell culture incubator, 37°C, 5% CO_2_
15‐ml centrifuge tubes, sterile (e.g., Corning, cat. no. 05‐538‐53F)
Centrifuge capable of ≥300 × *g* with holders for 15‐ml tube (e.g., Thermo Fisher, cat. no. 75007200)
Automated or manual cell counter
96‐well, ultralow‐attachment, U‐bottom plates (e.g., Sigma‐Aldrich, cat. no. CLS7007‐24EA)
6‐well, ultralow‐attachment plates (e.g., Corning cat. no. CLS3471)
Orbital shaker

#### Embryoid body formation (day –1)

1 When iPSCs are ready to passage, remove culture medium, and wash once with 2 ml DPBS per well for a 6‐well plate or 10 ml DPBS for a 10‐cm dish.
2 Add 0.5 ml Accutase to each well, and incubate 5 min at 37°C with 5% CO_2_.
  Prepare 10‐ml aliquots of Accutase, and store at −20°C. Thaw overnight at −4°C before use.
3 Transfer dissociated cells into a sterile 15‐ml centrifuge tube, and add three times the amount of prewarmed E8 medium.
  For example, if one well is dissociated with 0.5 ml Accutase, add 1.5 ml prewarmed E8 medium for a total of 2 ml.
  Prepare sufficient aliquots of E8 medium for 2 weeks’ worth of culture, and store aliquots at −20°C. Thaw aliquots the night before use at 4°C.
4 Centrifuge 4 min at 200 × *g*, room temperature.
5 Remove supernatant without disturbing the pellet, and resuspend pellet in 1 ml prewarmed E8 medium. Count cells with your method of choice.
6 Add prewarmed E8 supplemented with 10 µM ROCK inhibitor (1:1000 [v/v] dilution) to obtain a cell concentration of 100,000 cells/ml.
  Prepare 30‐μl aliquots of ROCK inhibitor, and store at −80°C.
7 Distribute 100 µl cell suspension in each well of a 96‐well ultralow‐attachment, U‐bottom plate using a multichannel pipette.
  This will result in initial embryoid bodies of 10,000 cells. The number of wells can be scaled up or down depending on the number of organoids to obtain.
8 Incubate at 37°C with 5% CO_2_, and leave undisturbed for 24 hr.

#### Neural induction (days 0 to 24)

9 Remove 80 µl medium using a multichannel pipette.
10 Replace with 100 µl prewarmed E6 medium supplemented with 2.5 µM dorsomorphin and 10 µM SB431542.
  Prepare sufficient aliquots of E6 medium for 2 weeks’ worth of culture, and store aliquots at −20°C. Thaw aliquots the night before use at 4°C.
11 Repeat steps 9 and 10 every day until day 6.
12 On day 6, transfer four to eight organoids to each well of a 6‐well, ultralow‐attachment plate. To transfer organoids without destroying them, cut the tip of a 1000‐µl pipette tip, and use it to transfer organoids individually.
13 Remove culture medium that was transferred with the organoids, and replace with prewarmed neural medium supplemented with 20 ng/ml bFGF and 20 ng/ml EGF.
14 Place plate on an orbital shaker at 65 rpm inside a 37°C, 5% CO_2_ incubator.
15 Replace medium daily from days 7 to 15 and then every other day from days 16 to 24.

#### Neural differentiation (from day 25)

**Figure 2:**
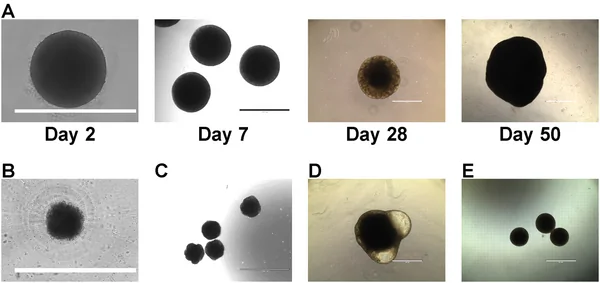
Brightfield images of developing cerebral organoids. (**A**) Normally developing cerebral organoids. (**B**) Cell aggregates at day 2 showing failure of embryoid body formation. (**C**) Abnormally small cerebral organoids at day 7. Note the abnormal irregular shape and lack of clear borders. This suggests abnormal differentiation of the organoids. (**D**) Presence of optically clear vesicles in a cerebral organoid suggesting the development of cyst‐like structures. (**E**) Fusion of two cerebral organoids. Fused organoids are usually easy to separate with gentle pipetting at this stage. Scale bars = 1 mm.

16 Starting on day 25, replace medium every other day with neural medium supplemented with 20 ng/ml BDNF and 20 ng/ml neurotrophin‐3.
17 On day 43, replace medium with neural medium without growth factor supplements. Change medium every 3 to 4 days (twice a week) until the desired time point.
  If the medium in some wells becomes too acidic (yellow coloration due to phenol red in the medium), decrease the number of organoids per well or change the medium more frequently. Organoids may fuse during culture. Fusion does not impair organoid development but reduces the total number of organoids obtained. To avoid this, decrease the number of organoids per well, and make sure to gently swirl the plate to distribute organoids within the well before placing the plate back in the incubator.
  Figure 2A shows an example of successful cerebral organoid formation.

## Support Protocol 1 HUMAN PLURIPOTENT STEM CELL CULTURE

Human PSCs are the starting material for human cerebral organoids. PSCs can be derived from established embryonic stem cell lines (e.g., H9 cell line) or obtained via reprogramming of somatic cells (e.g., fibroblasts), using different methods that are outside of the scope of this article, to generate iPSCs. The use of iPSCs to prepare human cerebral organoids is particularly interesting to investigate the effect of a compound on cells with a specific genetic mutation. The quality of PSCs and their pluripotency potential (ability to generate every cell type in an organism) is critical to successfully obtaining cerebral organoids. Indeed, if the stem cells used have already started to differentiate into any specific lineage, then some intermediate neural precursors may not be observed, or cerebral organoids may not form. Of note, PSCs can lose their pluripotency during culture through a variety of factors such as cellular stress or mycoplasma infection, among other ways. This protocol describes the culture of human iPSCs using a feeder‐free system (no layer of feeder cells such as mouse embryonic fibroblasts) and the maintenance of their pluripotency.

### Materials

Geltrex LDEV‐free reduced growth factor basement membrane matrix (e.g., Thermo Fisher Scientific, cat. no. A1413201) or Corning Matrigel hESC‐qualified matrix (e.g., Sigma‐Aldrich, cat. no. CLS354277)
Dulbecco's modified Eagle medium (DMEM)
Human iPSCs (e.g., ATCC, cat. no. BXS‐0117)
E8 medium (e.g., Stemcell Technologies, cat. no. 05990)
ROCK inhibitor Y27632 (e.g., Selleckchem, cat. no. S1049)
DPBS, without calcium or magnesium (e.g., Thermo Fisher Scientific, cat. no. 14190144)
0.5 mM EDTA (e.g., Wisent Bioproducts, cat. no. 325‐060‐EL)
Dimethyl sulfoxide (DMSO; e.g., Thermo Fisher Scientific, cat. no. AAJ66650AD)

6‐well plate, culture treated (e.g., Corning, cat. no. CLS3516) or 10‐cm dish
Cell culture incubator, 37°C, 5% CO_2_
37°C bead bath
15‐ml conical tubes, sterile (e.g., Corning, cat. no. 05‐538‐53F)
Centrifuge capable of ≥300 × *g* with holders for 15‐ml tube (e.g., Thermo Fisher, cat. no. 75007200)
Cryotubes
Freezing container (e.g., Mr. Frosty)
Liquid nitrogen tank

#### Thawing iPSCs

1 Coat two wells of a 6‐well plate or a 10‐cm dish with Geltrex or Matrigel. To do so, thaw a 60‐µl aliquot of Geltrex or Matrigel at 4°C. Place aliquot on ice, and dilute 60 µl in 3 ml cold DMEM (1:100 [v/v] final). Transfer 1 ml solution into each well of a 6‐well plate, or transfer 4 ml into a 10‐cm dish. Incubate plates/dishes at 37°C for 1 hr. Use immediately, or store at 4°C for up to 1 week.
2 Thaw a vial of iPSCs in a 37°C bead bath until a small piece of ice remains in the vial.
3 Transfer vial contents to a 15‐ml conical tube. Add 9 ml warmed E8 medium, and centrifuge 5 min at 200 × *g*, room temperature.
4 Remove supernatant without disturbing the pellet, and resuspend in 4 ml E8 medium supplemented with 10 µM ROCK inhibitor (1:1000 [v/v] dilution).
5 Aspirate Geltrex/Matrigel solution from coated wells, and transfer 2 ml cell suspension to each well.
6 Incubate at 37°C, 5% CO_2_.
7 Replace E8 medium every day without adding ROCK inhibitor.
  Colonies should be tightly packed with clear borders. Borders can initially appear “spiky” when cultured with ROCK inhibitor but should improve over 3 to 4 days.

#### Passaging iPSCs

Passaging should be performed when colonies are large and in close proximity, every ∼4 to 5 days.

8 Coat an appropriate number of wells with Geltrex or Matrigel.
9 Remove culture medium, and wash cells once by adding and removing 2 ml DPBS per well for a 6‐well plate or 10 ml DPBS for a 10‐cm dish.
10 Add 1 ml of 0.5 mM EDTA, and incubate for 5 min at 37°C.
  Colonies should start to round up and have small cell‐free areas inside them where cells have already detached.
11 Carefully remove EDTA using a pipette without detaching the cells, and add 2 ml E8 medium supplemented with 10 µM ROCK inhibitor to each well. Pipette up and down briefly to detach cells, avoiding the creation of bubbles.
  If cells have substantially detached before removing EDTA, incubate at room temperature. If cells do not detach with gentle pipetting or if a lot of cells are still attached, increase incubation time to ≥7 min. The goal is to obtain clusters of cells, not a single‐cell suspension.
12 Divide obtained cell solution at a 1:3 to 1:4 ratio into Geltrex‐coated plates/dishes with E8 medium supplemented with 10 µM ROCK inhibitor. Alternatively, proceed to step 14 to cryopreserve cells.
13 Incubate at 37°C, 5% CO_2_. Change medium the next day (without ROCK inhibitor).

#### Cryopreservation of iPSCs

14 After incubating cells with EDTA, remove EDTA and add 2 ml E8 medium to each well, pipetting up and down briefly to detach cells.
15 Transfer solution to a 15‐ml conical tube, and centrifuge 5 min at 200 × *g*, room temperature.
16 Remove supernatant and resuspend in E8 medium supplemented with 10% (v/v) DMSO.
  Add a volume equal to the number of wells that could have been seeded if the cells were simply passaged; for example, if you want to freeze the contents of one well of a 6‐well plate, resuspend in 3 ml to achieve a 1:3 split ratio.
17 Transfer 1‐ml aliquots to cryotubes.
18 Transfer cryotubes to a freezing container at −80°C, and after 24 hr transfer to a liquid nitrogen tank.
  For information about sample data, see Understanding Results.

## Basic Protocol 2 EVALUATION OF NEUROGENESIS IN CEREBRAL ORGANOIDS WITH ETHYNYL DEOXYURIDINE LABELING

Some neurotoxic compounds can cause brain malformations by altering the formation of new neurons during brain development, a process known as neurogenesis. New neurons are formed from neural stem cells that have the ability to differentiate into neurons and proliferate for self‐renewal (Kriegstein & Alvarez‐Buylla, 2009). Maintenance of an appropriate number in this pool of neural stem cells is critical to normal brain development because dysregulation of neural stem cell proliferation and differentiation results in abnormal brain function and is the cause of several neurological disorders (Duy et al., 2022; Krahe et al., 2015; Lancaster et al., 2013; Lipton & Sahin, 2014; Wang et al., 2020). Therefore, evaluating the effect of compounds on neurogenesis is an important step in evaluating their neurotoxicity. Neurogenesis can be assessed in organoids by “tagging” proliferating cells using a thymidine analogue such as EdU and determining their differentiation at a later time point using coimmunostaining with cell‐type specific markers. This protocol describes the different steps from incubating organoids with EdU (“pulse”) and evaluating neurogenesis after a desired period of time (“chase”). The duration of the pulse and chase can be manipulated to address different research questions related to the rate of proliferation and duration of cell cycle (short chase period) or the fate of the tagged cells (longer chase period).

### Materials

Click‐iT EdU cell proliferation kit (e.g., Thermo Fisher Scientific, cat. no. C10337)
Neural medium (see recipe)
Cerebral organoids (see Basic Protocol 1)
DPBS, without calcium or magnesium (e.g., Thermo Fisher Scientific, cat. no. 14190144)
4% (w/v) paraformaldehyde (e.g., Sigma‐Aldrich, cat. no. 1004960700)
30% (w/v) sucrose (e.g., Sigma‐Aldrich, cat. no. SLHV013SL)
Embedding solution (see recipe)
Ethanol/dry ice slurry
Triton X‐100 (e.g., Fisher Scientific, cat. no. BP151‐100)
Bovine serum albumin (BSA; e.g., Sigma‐Aldrich, cat. no. A3059)
10 mg/ml Hoechst 33342, trihydrochloride, trihydrate (e.g., Thermo Fisher Scientific, cat. no. H3570)

37°C incubator
1.5‐ml microcentrifuge tubes
Disposable embedding molds, square (e.g., Fisher Scientific, cat. no. 22‐363‐552)
Cryostat
Microscope slides
Hydrophobic pen (e.g., Fisher Scientific, cat. no. FSTP9778445)

#### EdU incorporation

1 Prepare 10 mM EdU by following the kit manufacturer's instructions.
2 Dilute 10 mM EdU into prewarmed neural medium to obtain a 40 µM EdU solution.
  Prepare a volume equal to half the culture medium of the organoids.
3 At the selected time point, remove half the medium from organoids in each well, and add an equal amount of 40 μM EdU to obtain a 20 µM EdU solution.
  Replacing half the medium helps prevent the temporary decrease in cell proliferation and therefore EdU incorporation seen with complete medium change.
4 Incubate organoids for 2 hr at 37°C.
5 Remove medium containing EdU, and wash organoids twice with prewarmed DPBS.
6 Add neural medium without EdU, and continue incubating organoids as usual.
  The length of this chase period depends on the research questions. When investigating proliferation, organoids can be processed for imaging immediately after the 2‐hr EdU pulse. If investigating changes in differentiation, then the chase should be continued until EdU‐tagged proliferating cells have differentiated, which can take several weeks. However, the longer the chase, the fainter the EdU signal will be because EdU will be diluted with each DNA replication.

#### Cryopreservation of organoids

7 At the selected time point after EdU pulse, wash organoids with DPBS.
8 Using a cut 1000‐µl pipette tip, transfer each organoid to a 1.5‐ml microcentrifuge tube containing 0.5 ml of 4% (w/v) paraformaldehyde.
9 Incubate at 4°C overnight.
10 Remove paraformaldehyde and perform three 15‐min washes with cold DPBS.
11 Remove DPBS and add 1 ml of 30% (w/v) sucrose to each microcentrifuge tube.
  This step is performed to dehydrate the organoids before freezing.
  Prepare 30% (w/v) sucrose by mixing 30 g sucrose in 100 ml deionized water. Store at 4°C for up to 2 weeks. If longer storage is desired, the solution can be filter sterilized using a 0.45‐µm syringe membrane filter.
12 Incubate at 4°C until organoids have sunk to the bottom of the tube (usually overnight).
13 Fill disposable square molds with embedding solution.
14 Using a 1000‐µl pipette with a cut tip, transfer organoids to the mold, making sure to transfer as little 30% (w/v) sucrose as possible.
15 Prepare ethanol/dry ice slurry by adding 100% ethanol to dry ice pellets, and snap freeze organoids by placing the molds in the ethanol/dry ice slurry.
16 Keep at −80°C until sectioning.

#### Immunofluorescence staining

17 Cut sections using a cryostat set at −20°C, and place sections on a microscope slide.
  Sections can be up to 20 µm thick if using a confocal microscope; 10‐µm sections can be used if using an epifluorescence microscope. Sections can be stored indefinitely at −80°C.
18 Delineate an area around each section with a hydrophobic pen.
19 Wash each section once with DPBS to remove the optimal cutting temperature (OCT) compound contained in the embedding solution.
20 Permeabilize sections by incubating with 0.5% (v/v) Triton X‐100 in DPBS for 20 min at room temperature.
21 Remove Triton X‐100, and wash sections twice with 3% (w/v) BSA in DPBS.
22 Prepare Click‐iT EdU reaction cocktail according to manufacturer's instruction. Remove 3% (w/v) BSA, and cover section with the reaction cocktail.
  All reagents need to be at room temperature, and the order of reagent addition is important for a successful reaction.
23 Incubate for 30 min at room temperature, protected from light.
24 Remove reaction cocktail, and wash once with 3% (w/v) BSA in DPBS.
25 Incubate 10 min with Hoechst 33342 (diluted 1:5000 [v/v] in DPBS) to counterstain nuclei, or proceed with labeling using primary antibodies (Support Protocol 2).
  For information about sample data, see Understanding Results.

## Basic Protocol 3 CALCIUM IMAGING IN CEREBRAL ORGANOIDS

Neurotoxicity of compounds may affect neuronal excitability, which is critical for normal brain function. After stimulation of neurons by an excitatory neurotransmitter, depolarization of the stimulated neurons follows, which in turn triggers calcium influx into the depolarized neurons. This calcium signaling reflects neuronal activity both *in vitro* and *in vivo*. To measure calcium signaling in real time, neurons can be incubated with fluorescent calcium indicators that undergo an increase in fluorescence when binding calcium. By measuring this calcium‐associated fluorescence in real time with a fluorescence microscope, calcium imaging allows researchers to follow neuronal activity at a single‐cell resolution and assess the effect of certain compounds on neuronal activity. This protocol describes the use of a fluorescent calcium indicator, Fluo‐4 AM, with cerebral organoids to measure changes in neuronal activity induced by 4‐aminopyridine (4‐AP), a potassium channel blocker. The data acquired by following this protocol will enable the investigator to characterize changes in calcium signaling such as changes in transient amplitude, frequency, and synchronization.

### Materials

DMSO (e.g., Thermo Fisher Scientific, cat. no. AAJ66650AD)
Fluo‐4 AM, 50‐μg vial (e.g., Thermo Fisher Scientific, cat. no. F14201)
20% (w/v) Pluronic F‐127 in DMSO (e.g., Thermo Fisher Scientific, cat. no. P3000MP)
Neural medium (see recipe)
Cerebral organoids (see Basic Protocol 1)
Artificial cerebrospinal fluid (aCSF; see recipe)
4‐AP (e.g., Sigma‐Aldrich, cat. no. 275875)

Vortex mixer
35‐mm glass‐bottom culture dish (e.g., Thermo Fisher Scientific, cat. no. 150680)
Cell culture incubator, 37°C, 5% CO_2_
Inverted fluorescence microscope or confocal microscope (e.g., Olympus FV1000) with 488‐nm excitation laser and FITC filter
Compute running FIJI (ImageJ; available at: https://imagej.nih.gov/ij) and statistical analysis software

#### Fluo4‐AM loading in organoids

1 Add 44 µl DMSO to a 50‐µg vial of Fluo‐4 AM to make 1 mM Fluo‐4 AM. Vortex for 1 min.
  Fluo‐4 AM should not be exposed to light. Work quickly in the dark. The solution can be stored in the dark at −20°C for several weeks.
2 Aliquot the volume of reconstituted Fluo‐4 AM to be used for the experiment (e.g., 4 µl Fluo‐4 AM per 35‐mm dish). Add an equal amount of 20% (w/v) Pluronic F127, and vortex for 1 min.
3 Mix 4 μl Fluo‐4 AM/Pluronic F127 and 1 ml prewarmed neural medium to prepare 2 µM Fluo‐4 AM/Pluronic F127. Prepare 2 ml per 35‐mm dish needed. Vortex for 1 min.
4 Incubate organoids in Fluo‐4 AM/culture medium in a 35‐mm dish at 37°C, 5% CO_2_ in the dark.
5 Wash organoids once with prewarmed aCSF, and incubate at 37°C, 5% CO_2_ for 20 min.
  This ensures that Fluo‐4 AM that has not permeated the cell is removed, which decreases the background fluorescence intensity.

#### Calcium imaging

6 Transfer organoids onto the stage of a confocal microscope for imaging, and focus on the region of interest (ROI).
  If organoids drift excessively in the field, remove some aCSF so that organoids are immobile on the bottom of the dish. Make sure to leave enough aCSF to cover the organoids and prevent drying.
7 Acquire a timed exposure series by taking images with 488‐nm laser excitation and FITC filter at a set frequency.
  The frequency depends on the process under study. Ideally, a sampling frequency twice the frequency of the calcium waves under study is needed to allow sufficient temporal resolution to capture the kinetics of the calcium waves. A slower frequency allows for better image resolution but worse time resolution. Calcium waves triggered by neuronal excitation and detected by Fluo‐4 AM are several seconds long, and therefore a frequency of 2 Hz is acceptable. The length of the time series is determined by individual experimental needs and the available memory of the computer running the microscope software. Recordings of 3 to 5 min are usually performed. An example of a calcium imaging recording is given in Video S1 (in Supporting Information).
8 After acquiring the time series pre‐excitation, replace aCSF with prewarmed aCSF containing 100 µM 4‐AP. Incubate for 5 min at 37°C, 5% CO_2_ in the dark.
9 Repeat acquisition of the time series.

#### Data processing in FIJI

10 Open time series with FIJI.
  Tutorials to install and use basic ImageJ functions are available at https://imagej.nih.gov/ij/docs/guide/user‐guide.pdf and https://imagej.net.
11 Make a *z*‐projection using FIJI (Image → Stacks → Z Project) using the maximum standard deviation to identify responsive and unresponsive cells.
12 Trace an ROI around the cell body of individual neurons and a cell‐empty area for background measurements using the freehand or polygon selection tools.
13 Add ROI to the measurements by using the ROI manager tool (Analyze → Tools → ROI Manager).
14 Save ROI and export ROI on the time series.
15 Measure mean intensity for each ROI on each image (More → Multi‐measure).
  This will give you a matrix with each column representing each neuron (as well as one column for the background) and each row representing a time point.
16 Export and analyze with statistical software.
  Examples of statistical software include R, MATLAB, GraphPad Prism, Python, and Excel.
17 Correct for background fluorescence by subtracting the background intensity for each neuron at each time point.
18 Calculate relative change in fluorescence (ΔF) for each neuron for each frame using the equation:
$$\Delta F\;=\;\frac{(F\;-\;F_{\min})}{F_{\min}}$$
  where F represents the mean fluorescence of a neuron at a particular time and F_min_ represents the minimum fluorescence of that neuron.
  Alternatively, if the organoids are stimulated, use the intensity before stimulation (F_0_) instead of the minimum intensity (F_min_). ΔF can be plotted over time, and individual neuron peak frequency, amplitude, and duration can be determined before and after stimulation by 4‐AP depending on the research question.
  Programs allowing automated analysis of calcium imaging are also available (Giovannucci et al., 2019; Pnevmatikakis, 2019; Romano et al., 2017).
  For information about sample data, see Understanding Results.

## Basic Protocol 4 ELECTROPHYSIOLOGICAL EVALUATION OF CEREBRAL ORGANOIDS WITH MICROELECTRODE ARRAYS

To assess the neurotoxicity of a compound, it is often necessary to study its effect on neuronal activity. As seen in Basic Protocol 3, neuronal activity can be measured at the single‐cell level with calcium imaging. Despite its high spatial resolution, calcium imaging typically only records the activity of a limited number of cells and with a low time resolution (around 2 Hz). Conversely, acquiring electrophysiological recordings of neuronal local field potentials with MEAs offers high time resolution (up to 12.5 kHz) and the ability to record different regions of the organoids. This allows for the measurement of a compound's effects on neuronal network connectivity within the organoid by assessing synchronization of activity recorded by different electrodes. MEA recording therefore offers a complementary approach to calcium imaging. As organoids can be grown directly onto the MEA or placed on the MEA before recording, longitudinal recordings throughout organoid maturation can be performed. This protocol describes the recording of cerebral organoids with MEAs using the Axion Maestro Edge MEA system. Data obtained through this protocol are typically analyzed by software such as MATLAB or the integrated AxIS software.

### Materials

Poly‐l‐ornithine (e.g., Sigma‐Aldrich, cat. no. P4957)
1 mg/ml laminin (e.g., Sigma‐Aldrich, cat. no. L2020‐1MG)
DPBS, without calcium or magnesium (e.g., Thermo Fisher Scientific, cat. no. 14190144)
Cerebral organoids (see Basic Protocol 1)
Neural medium (see recipe)
4‐AP (e.g., Sigma‐Aldrich, cat. no. 275875)

6‐well MEA plate (e.g., CytoView MEA Plate; Axion Biosystems, cat. no. M384‐tMEA‐6W)
Maestro Edge 384‐channel system (Axion Biosystems)
Cell culture incubator, 37°C, 5% CO_2_
Computer running AxIS software (Axion Biosystems)

1 Add 0.5 ml poly‐l‐ornithine to each well of a recording plate to cover the electrode array. Incubate at 37°C for 4 hr or at 4°C overnight.
2 Dilute 1 mg/ml laminin in cold DPBS to obtain a 20 µg/ml solution (1:50 dilution). Aspirate poly‐l‐ornithine, and add 0.5 ml diluted laminin to cover the electrode arrays. Incubate at 37°C for 4 hr or at 4°C overnight.
  Always dilute laminin in ice‐cold DPBS to prevent gelling of the solution. Unused laminin can be aliquoted undiluted and kept at −20°C. To use, thaw laminin slowly on ice to prevent gelling.
3 Remove laminin. Using a 1000‐µl pipette with a cut tip, place organoid on the MEA.
  Do not let the laminin solution dry before adding the organoid. Laminin should be removed just before organoid transfer in each well. Add the organoid in a single drop of culture medium centered on the electrode array. This will ensure that the organoid will adhere to the electrode array. If coated plates are not used right away, they should be sealed with Parafilm and stored at 4°C for up to 1 week.
4 Add sterile distilled water to the plate in between wells. Incubate plates with organoids at 37°C, 5% CO_2_ overnight.
  Adding water will decrease evaporation of the culture medium.
5 The next day, carefully add 1 ml prewarmed neural medium.
  Organoids can easily detach from the array. Add the medium very carefully using a pipette.
6 Replace medium as usual, handling plates with care so as not to detach organoids.
  Younger organoids (<4 weeks old) will adhere better than older organoids (>8 weeks old). Organoids can therefore be seeded well in advance of recording and cultured in an adherent fashion in the MEA plates. If organoids detach, recording is still possible. To ensure maximal contact between the organoid and the MEA, medium can be removed leaving only around 200 µl to prevent drying of the organoid during the recording session. Otherwise, an electrophysiology slice anchor (harp) can also be put on top of the organoids during the recording session, although some damage to the organoid would be expected.
7 At the selected time points, place MEA plate inside the Maestro Edge recording chamber.
8 Open AxIS software, and record in spontaneous neural configuration.
  The length of recording sessions varies between experiments, but a good starting point is 5 to 10 min.
9 Once the baseline recording is acquired, add 4‐AP to a final concentration of 100 µM, and incubate plate at 37°C, 5% CO_2_ for 5 min.
10 Repeat recording with the same parameters.
  Recorded files can be analyzed using the Axion Biosystems Neural Metrics Tool or other signal‐processing software such as MATLAB or Python. Dedicated open‐source toolboxes have also been developed by other laboratories (Egert et al., 2002; Pastore et al., 2016).

## Support Protocol 2 IMMUNOSTAINING OF CEREBRAL ORGANOIDS

Immunostaining is a common technique employed with cerebral tissue that can be applied to cerebral organoids. It allows for visualization of the location of an antigen (usually a peptide) by using specific antibodies binding to the antigen. In the case of fluorescence immunostaining, as described in this protocol, the antibodies are either bound to a fluorophore, or secondary fluorophore‐bound antibodies are used to reveal the presence of the primary antibodies. By using antibodies binding to a peptide specific for a cell type (e.g., microtubule‐associated protein 2 [MAP2] for neurons, SRY‐box transcription factor 2 [SOX2] for neuroepithelial cells) or a phenotype (e.g., cleaved‐caspase 3 for apoptotic cells), the proportion of different cell populations can be quantified and compared between treated and control organoids. Fluorescence immunostaining can also be used to characterize the spatial localization of a specific peptide within a cell. In this protocol, we describe the steps to perform fluorescence immunostaining with cerebral organoids. This protocol can be used with Basic Protocol 2, which describes EdU staining in cerebral organoids. The combination of these protocols will allow researchers to assess the differentiation of EdU‐tagged cells into different cell types.

### Materials

Cerebral organoids (see Basic Protocol 1)
DPBS, without calcium or magnesium (e.g., Thermo Fisher Scientific, cat. no. 14190144)
4% (w/v) paraformaldehyde (e.g., Sigma‐Aldrich, cat. no. 1004960700)
30% (w/v) sucrose
Embedding solution (see recipe)
Ethanol/dry ice slurry
Sodium citrate buffer (see recipe)
Blocking buffer (see recipe)
Primary antibodies for protein of interest
Antibody dilution buffer (see recipe)
Alexa Fluor–conjugated secondary antibodies
DAPI
Antifade mounting medium (e.g., VECTASHEILD; Vector Laboratories, cat. no. H120010)
Clear nail polish

1.5‐ml microcentrifuge tubes
Disposable embedding molds (e.g., Fisher Scientific, cat. no. 22‐363‐552)
Cryostat (e.g., Leica CM1950)
Microscope slides
Hydrophobic pen (e.g., Fisher Scientific, cat. no. FSTP9778445)
Coplin jar
95°C vegetable steamer
Humidified chamber (e.g., plastic pipette tip box containing a damp tissue)
Inverted fluorescence microscope or confocal microscope (e.g., Olympus FV1000)

#### Cryopreservation of organoids

1 At the selected time point, wash organoids with DPBS.
2 Using a 1000‐µl pipette tip with cut tip, transfer each organoid to a 1.5‐ml microcentrifuge tube containing 0.5 ml of 4% (w/v) paraformaldehyde.
3 Incubate at 4°C overnight.
4 Remove paraformaldehyde and perform three 15‐min washes with cold DPBS.
5 Remove DPBS and add 1 ml of 30% (w/v) sucrose to each microcentrifuge tube.
  This step is performed to dehydrate the organoids before freezing.
6 Incubate at 4°C until organoids have sunk to the bottom of the tube.
7 Fill disposable embedding molds with embedding solution.
8 Using a 1000‐µl pipette with cut tip, transfer organoids to the mold, making sure to transfer as little 30% (w/v) sucrose as possible.
9 Snap freeze organoids by placing molds in ethanol/dry ice slurry.
10 Keep at −80°C until sectioning.

#### Organoid section, permeabilization, and blocking

11 Cut sections using a cryostat set at −20°C, and place sections on a microscope slide.
  Sections can be up to 20 µM thick if using a confocal microscope; 10‐µM sections can be used if using an epifluorescence microscope. Sections can be stored indefinitely at −80°C.
12 Delineate an area around each section with a hydrophobic pen. Let ink dry completely.
13 Wash each section once with DPBS to remove the OCT compound contained in the embedding solution.
14 *Optional*: Perform antigen retrieval by incubating slides with the section in a Coplin jar filled with sodium citrate buffer for 20 min in a vegetable steamer at 95°C.
  This step facilitates the removal of antigen masking, which is caused by paraformaldehyde crosslinking with proteins. It is not necessary for all antigens but is required for others such as Ki67.
15 Permeabilize and block sections by incubating with blocking buffer for 1 hr at room temperature.

#### Primary antibody incubation

16 Remove blocking buffer, and replace with primary antibodies diluted in antibody dilution buffer.
  Concentration of the primary antibody depends on the antibody and specific samples. The optimal dilution needs to be determined empirically beforehand.
17 Place slides in a humidified chamber, and incubate overnight at 4°C.
  A humidified chamber is used to avoid drying of the sections.

#### Secondary antibody incubation and counterstaining

18 Remove primary antibody, and wash sections three times for 10 min per wash with antibody dilution buffer at room temperature.
19 Cover section with secondary antibody diluted in antibody dilution buffer.
  The concentration of the secondary antibody also needs to be determined empirically. As the secondary antibodies are conjugated to an Alexa Fluor, this and the following steps need to be performed in the dark.
20 Incubate for 2 hr at room temperature protected from light in a humidified chamber.
21 Remove secondary antibody, and wash sections three times for 10 min per wash with antibody dilution buffer.
22 Counterstain with DAPI diluted in DPBS for 5 min at room temperature.
23 Remove DAPI and wash once with DPBS for 5 min at room temperature.
24 Apply a drop of antifade mounting medium onto each section, and cover with a coverslip.
25 Seal coverslip with nail polish.
26 Store slides at 4°C protected from light.
27 Acquire images using a fluorescence or confocal microscope equipped with lasers and filters that match the Alexa Fluor fluorophores used.

## REAGENTS AND SOLUTIONS

### aCSF

480 ml double distilled water
4.24 g NaCl (145 mM final)
0.09 g KCl (2.5 mM final)
0.11 g CaCl_2_ (2 mM final)
1.19 g HEPES (10 mM final)
0.9 g dextrose (10 mM final)
0.05 g MgCl_2_ (1 mM final)
50 µl of 10,000× glycine (2 µM final)
Adjust pH to 7.3 with HCl
Bring volume to 500 ml with double distilled water
Adjust osmolarity to 315 to 325 mOsm with sucrose
Use immediately or filter with a 0.45‐µm filter unit and store at 4°C for up to 2 weeks
  To prepare 10,000× glycine, dilute 15 mg glycine in 10 ml double distilled water.

### Antibody dilution buffer

100 ml deionized water
0.5 g BSA (0.5% [w/v] final)
200 µl Triton X‐100 (0.2 % [w/v] final)
Store at 4°C for up to 1 week

### BDNF

DPBS (e.g., Thermo Fisher Scientific, cat. no. 14190144)
100 μg/ml BDNF (e.g., Stemcell Technologies, cat. no. 78005)
0.1% (w/v) BSA
Prepare 20‐µl aliquots
Store at −80°C for up to 6 months
  Once thawed, store at 4°C, and use within 2 weeks.

### bFGF

100 μg/ml bFGF (e.g., R&D Systems, cat. no. 233‐FB‐025)
0.1% (w/v) BSA
DPBS (e.g., Thermo Fisher Scientific, cat. no. 14190144)
Prepare 20‐µl aliquots
Store at −80°C for up to 6 months
  Once thawed, store at 4°C, and use within 2 weeks.

### Blocking buffer

100 ml deionized water
1 g BSA (1% [w/v] final)
300 µl Triton X‐100 (0.3% [v/v] final)
Store at 4°C for up to 1 week

### Dorsomorphin, 5 mM

5 mg dorsomorphin (e.g., Sigma‐Aldrich, cat. no. P5499)
2.5 ml sterile DMSO
Store 60‐µl aliquots at −80°C for up to 6 months
  Once thawed, store aliquots at 4°C, and use within 2 weeks.

### EGF

DPBS (e.g., Thermo Fisher Scientific, cat. no. 14190144)
100 μg/ml EGF (e.g., R&D Systems, cat. no. 236‐EG‐20)
0.1% BSA
Prepare 20‐µl aliquots
Store at −80°C for up to 6 months
  Once thawed, store at 4°C, and use within 2 weeks.

### Embedding solution

Mix equal parts 30% (w/v) sucrose with OCT compound (e.g., Tissue‐Tek, cat. no. 4583). Shake until both components have mixed, and let rest at 4°C until all bubbles have disappeared. Store at 4°C for up to 2 weeks.

### Neural medium

480 ml neurobasal medium (e.g., Thermo Fisher Scientific, cat no. 21103049)
10 ml B‐27 supplement minus vitamin A (2% [v/v] final; e.g., Thermo Fisher Scientific, cat. no. 12587010)
5 ml GlutaMax (1% [v/v] final; e.g., Thermo Fisher Scientific, cat. no. 35050061)
5 ml penicillin‐streptomycin, 10,000 U/L (1% [v/v] final; e.g., Thermo Fisher Scientific, cat. no. 15140122)
Filter using a 0.45‐µm filter unit (e.g., Thermo Fisher Scientific, cat. no. 169‐0045) and vacuum pump
Store at 4°C for up to 2 weeks
  To use, aliquot only the required amount in a 15‐ or 50‐ml conical tube, and warm to 37°C in a bead bath. Add growth factors and small molecules immediately before use.

### Neurotrophin‐3

DPBS (e.g., Thermo Fisher Scientific, cat. no. 14190144)
100 μg/ml neurotrophin‐3 (e.g., Stemcell Technologies, cat. no. 78074)
0.1% (w/v) BSA
Prepare 20‐µl aliquots
Store at −80°C for up to 6 months
  Once thawed, store at 4°C, and use within 2 weeks.

### SB431542, 10 mM

10 mg SB431542 (e.g., Tocris, cat. no. 1614‐10MG)
2.60 ml of 100% (v/v) ethanol
Store 120‐μl aliquots at −80°C for up to 6 months
  Once thawed, store at 4°C, and use within 2 weeks.

### Sodium citrate buffer

950 ml deionized water
2.1 g citric acid dihydrate
Adjust pH to 6 with HCl or NaOH
Add 0.5 ml Tween‐20
Bring to 1 L with deionized water
Store at 4°C for up to 6 months

## COMMENTARY

### Background Information

The term cerebral organoid encompasses the products of diverse 3D culture techniques that rely on the self‐organizing properties and codevelopment of the starting cells. The starting cells can either be PSCs or primary tissue stem cells depending on the type of neural structure to be obtained *in vitro* (Pașca et al., 2022). Human cerebral organoids have provided an invaluable resource for the investigation of brain development. Previous models to evaluate the toxic potential of a compound on the developing brain consisted of the use of laboratory rodents and cell cultures grown in a monolayer. These models had important limitations including interspecies differences and the lack of specific cell‐cell interactions in monolayer cultures. These limitations are at least partially compensated for by the use of human iPSCs to generate cerebral organoids and by the 3D organization of organoids that forms with an organization similar to the developing neural tube. By recapitulating the early, *in utero* stages of central system development, human cerebral organoids allow researchers to evaluate the neurotoxicity of a compound on a previously inaccessible window of human neurodevelopment. In a recent study, we used this approach to describe the effect of 4‐AP, a potassium channel blocker, on early neurogenesis in human cerebral organoids (Parmentier et al., 2022). Since the first description of cerebral organoids in mouse and humans, several protocols have been published both from embryonic stem cells and iPSCs (Birey et al., 2017; Eiraku et al., 2008; Giandomenico et al., 2019; Kadoshima et al., 2013; Lancaster et al., 2013; Paşca et al., 2015; Velasco et al., 2019; Yoon et al., 2019; Zafeiriou, Guobin, et al., 2020). Protocols differ mainly in the use of small molecules and growth factors to guide PSCs toward differentiating into regionally specific cell types (guided protocols) or approaches where PSCs progressively differentiate into the neuroectodermal lineage that is the default lineage in the absence of guiding signals (unguided protocols; Pașca et al., 2022). Guided protocols allow one to obtain cerebral organoids composed of cells representative of a specific nervous system region with high reproducibility, such as the dorsal forebrain, midbrain, or hypothalamus. Unguided protocols generate more diverse organoids with increased cell type diversity that can vary from organoid to organoid. The use of guided or unguided protocols should be based on the question asked. Guided protocols may provide more reproducible results in downstream assays but only test the compound on specific neural cell types.

**Figure 3:**
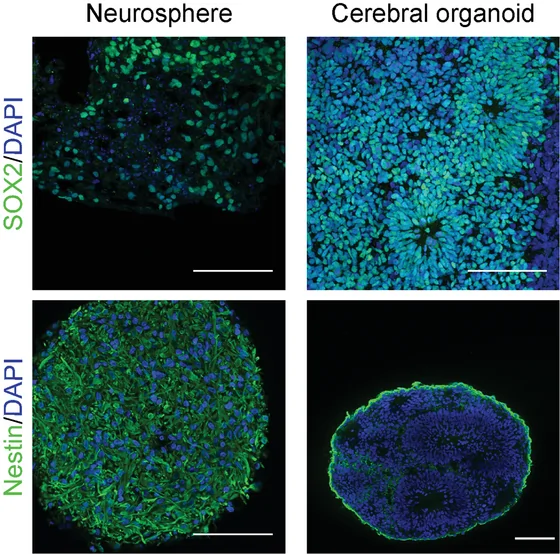
Differences in organization between neurospheres and cerebral organoids. Fluorescent immunohistochemical images of neurospheres and cerebral organoids after 18 days of culture. Cerebral organoids show neural tube–like structures comprising SRY‐box transcription factor 2 (SOX2)‐positive cells, whereas SOX2‐positive cells are not organized similarly in neurospheres. Neurospheres comprise an aggregate of nestin‐positive neural stem cells. Scale bars = 100 µm.

Despite the advantages offered by cerebral organoids to study the effect of chemicals on neurodevelopment, cerebral organoids remain a simplified model of brain development with several important differences between cerebral organoids and the brain. First, although cerebral organoids differ from neural cells grown in monolayers by their layered organization (Fig. 3), they lack clearly developed ventricles and distinct cortical layers observed in the brain. Second, using currently available protocols, cerebral organoids lack non‐neuroectodermal cells that form important brain structures such as meninges, blood vessels, and microglia. All of these cells participate in normal brain development. Furthermore, the lack of blood vessels in organoids contribute to necrosis of the organoid center as the structure grows because of a lack of nutrient and oxygen diffusion at its core (Cakir et al., 2019). Cerebral organoids display a remarkable neuronal cell diversity that closely matches the developing brain, although cellular stress triggered by the culture conditions has been shown to limit this cellular diversity with several neuronal subtypes incorrectly represented (Bhaduri et al., 2020; Quadrato et al., 2017; Velasco et al., 2019). Third, cerebral organoids closely recapitulate the early stages of brain development in terms of transcriptional profile, epigenomic signature, and electrical activity (Gordon et al., 2021; Luo et al., 2016; Trujillo et al., 2019). However, cerebral organoids are not as close to the fully mature brain in terms of complex electrical activity, and a divergence in transcriptional profile is observed between cerebral organoids and the postnatal brain (Gordon et al., 2021; Trujillo et al., 2019).

As cerebral organoid protocols progress and become more refined, highly specialized structures can be generated in cerebral organoids either by adding cells differentiated separately to cerebral organoids or by using PSCs and sequential patterning signals to differentiate stem cells into a specific brain region. As such, choroid plexus‐ and hippocampal‐like tissues have been generated directly from human PSC‐derived organoids *in vitro* (Pomeshchik et al., 2020; Sakaguchi et al., 2015). In contrast, to add vessel‐like structures to cerebral organoids or assess the role of microglia in brain development, these nonectodermal cells are differentiated separately and then added to cerebral organoids (Cakir et al., 2019; Xu et al., 2021). To allow better organoid perfusion and the establishment of long‐range connections between neurons, cerebral organoids can also be grafted into immunodeficient mice to provide a functional vascular system (Bhaduri et al., 2020). The protocols we describe here do not use any special equipment and are easily scalable. An important feature of cerebral organoids is that they recapitulate key aspects of brain development in terms of gene expression and neuronal activity (Gordon et al., 2021; Trujillo et al., 2019; Zafeiriou, Bao, et al., 2020). Although gene expression can be measured using techniques such as real‐time PCR, RNA sequencing, western blot, and immunostaining, we offer two simple protocols to assess neuronal activity with calcium imaging and MEAs. These functional assays are critical to evaluate the effect of a compound on neuronal activity.

### Critical Parameters

Cerebral organoids stand out from less‐complex models of human neurodevelopment, such as iPSC‐derived neurons grown in monolayers or neurospheres, by the recapitulation of key developmental aspects similar to the developing neural tube. The complex layered organization of cells in cerebral organoids is not observed in neurospheres generated from human neural stem cells (Lancaster & Knoblich, 2014). Moreover, neurospheres do not show the complex oscillatory activity recorded from cerebral organoids (Trujillo et al., 2019). One of the critical differences between the generation of cerebral organoids and neurospheres is the starting material—iPSCs and iPSC‐derived neural stem cells, respectively. To obtain cerebral organoids that recapitulate the early stages of development, starting iPSCs must have fully retained their pluripotent potential and not started the differentiation process. Premature differentiation of iPSCs will result in a lack of the neural tube–like zones and the typical layered organization of the organoids (Fig. 3).

Culture of cerebral organoids is a lengthy process taking several weeks to months. The appropriate time point when organoids should be used for downstream experiments is based on the developmental window being investigated. Early organoids (<30 days in culture) are mainly composed of proliferating neural precursors. Postmitotic neurons appear after 30 days and progressively acquire mature electrophysiological properties that can be observed starting around 4 months of culture. More complex, synchronized neuronal activity is observed after 6 months of culture. Glial cells start to appear between 2 and 3 months of culture (Parmentier et al., 2022; Sloan et al., 2018; Trujillo et al., 2019). By defining the targets of the neurotoxic compound, investigators can select the appropriate time points for their assays. It is therefore critical to maintain strict aseptic technique to avoid bacterial or fungal contamination resulting in sample loss. Moreover, routinely checking for mycoplasma contamination of the iPSC lines themselves, as well as during organoid culture, is recommended through the use of commercial PCR kits. Mycoplasma contamination can interfere with normal cell differentiation within cerebral organoids.

Many components of the neural medium, as well as growth factors used in this protocol, have limited stability. To help achieve optimal differentiation with low cell death, fresh media components are critical. Neural medium should be prepared and used within 2 weeks. Growth factors should be aliquoted as recommended and kept frozen until use.

Finally, although guided protocols for the generation of cerebral organoids such as the one presented here are fairly reliable (Yoon et al., 2019), variability between batches of cerebral organoids has been observed in downstream experiments. One of the main sources of variability is the iPSC lines used for organoid generation (Cahan & Daley, 2013; Hernández et al., 2022). To increase reproducibility of results and their generalization, cerebral organoids should be generated from multiple iPSC lines and in several batches for each experiment.

### Troubleshooting

See Table 1 for a troubleshooting guide regarding organoid generation and downstream applications.

**Table 1:** Troubleshooting Guide for Cerebral Organoid Generation and Downstream Applications

Problem	Possible cause	Solution
Disintegration of cerebral organoids	Contamination by infectious agents	Check culture for mycoplasma contamination; use strict aseptic technique
	Inactive ROCK inhibitor	Use fresh small molecules and growth factors
Improper neuronal differentiation	Loss of growth factor and small molecule activity	Use fresh small molecules and growth factors
Appearance of cysts or clear vesicles	Improper differentiation into neuroectoderm	Check pluripotency of starting iPSCs
		Use fresh small molecules and growth factors
Poor EdU staining signal	Improper Click‐iT reaction	Closely follow manufacturer's instruction regarding reagent temperature and mixing order
	Chase period is too long	Consider alternative fate‐mapping experiment (e.g., retroviral labeling)
Lack of activity in calcium imaging or electrophysiology	Improper neuronal differentiation	Check neuronal differentiation with immunostaining using neuronal markers
		Use fresh small molecules and growth factors
Cerebral organoids keep detaching from MEA grid	Improper electrode coating	Try different coatings such as laminin, poly‐l‐ornithine, or polyethylenimine
	Too much manipulation of the plate	Add medium carefully
		Place organoid on electrode grid just before recording; leave only minimal medium
		Place electrophysiology harp on top of organoid for stabilization

EdU, ethynyl deoxyuridine; iPSC, induced pluripotent stem cell; MEA, microelectrode array; ROCK, Rho‐associated protein kinase.

### Understanding Results

By following Basic Protocol 1, investigators should obtain cerebral organoids similar to the examples provided in Figure 2A. Embryoid bodies that successfully form are around 500 µm in diameter and have a well‐defined border (Fig. 2A, day 2). In contrast, failure to reaggregate will produce cell clumps with an undefined border (Fig. 2B). As cerebral organoids differentiate, they should progressively increase in size and reach 1 mm by weeks 3 to 4. The periphery of the organoids is usually brighter than the center when observed under a brightfield microscope with sometimes well‐defined neural tube–like structures (Fig. 2A, days 7 and 28). Cerebral organoids will usually reach several millimeters in diameter after weeks to months of culture. Failure to properly differentiate will result in organoids of small diameter with unclear borders and an absence of brighter periphery (Fig. 2C) or presence of cystic structures (Fig. 2D). Cerebral organoids may also fuse together, especially if their numbers are too high within a well (Fig. 2E). This may not impair their normal development but will reduce their final yield. It is therefore advisable to prepare a larger number of cerebral organoids than actually needed to compensate for the fusion of some organoids. Early on, during fusion (such as presented in Fig. 2E), it may be possible to separate fused organoids by pipetting gently with a 1000‐μl pipette tip that has been cut at the opening until separation is observed, taking care not to damage them.

**Figure 4:**
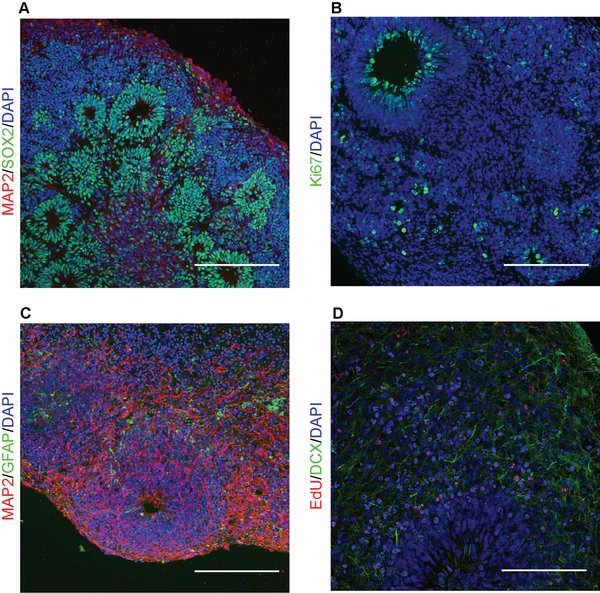
Neurogenesis and gliogenesis in cerebral organoids. Fluorescent immunohistochemical images of cerebral organoids at days 30 (**A** and **B**) and 120 (**C** and **D**). (A) Presence of numerous neural tube–like structures comprising SRY‐box transcription factor 2 (SOX2)‐positive neural precursors. Microtubule‐associated protein 2 (MAP2)‐positive neurons are present in between the neural tube–like structures and at the periphery of the organoids. (B) Actively dividing Ki67‐positive cells are present within the neural tube–like structures. (C) MAP2‐positive neurons and glial fibrillary acid protein (GFAP)‐positive glial cells are present after 120 days of culture. Neural tube–like structures may be smaller, be less numerous, or have disappeared at this time. (D) Evaluation of neurogenesis with ethynyl deoxyuridine (EdU) and doublecortin (DCX) coimmunostaining to evaluate differentiation of neural stem cells into neurons. Scale bars = 100 µm.

Successful cerebral organoid generation will result in organoids comprising circular neural tube–like structures composed of SOX2‐positive early neural precursors (Figs. 3 and 4A). These neural tube–like structures comprise actively proliferating cells that will progressively differentiate into neural stem cells (nestin‐positive), neuroblasts (doublecortin‐positive), neurons (MAP2‐positive), and glial cells (glial fibrillary acidic protein–positive; Fig. 4). Failure to observe these neural tube–like structures indicates improper recapitulation of the early stages of neurogenesis and is typical of neurospheres (Fig. 3). This usually indicates loss of pluripotency of the starting iPSCs. By following Basic Protocol 2 and Support Protocol 2, neurogenesis can be followed by tagging proliferating cells with EdU and observing their differentiation using cell type–specific markers and immunostaining (Fig. 4D). In cerebral organoids grown for ≥3 months and several millimeters in diameter, a necrotic area in the center of the organoid may develop due to limited diffusion of oxygen and nutrients.

**Figure 5:**
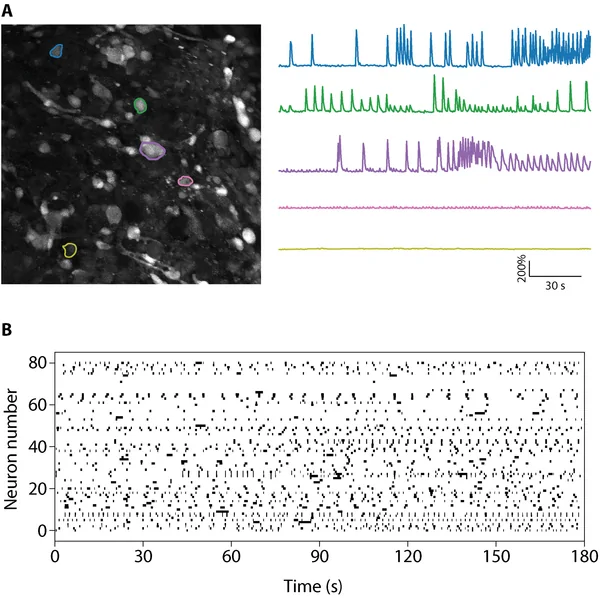
Calcium imaging in cerebral organoids. (**A**) Relative change in fluorescence over time of selected cells. (**B**) Raster plot of calcium events. Recording of an organoid at day 120.

Functional assays to evaluate neuronal activity in cerebral organoids are critical to assess the effect of a compound on neuronal function. As cerebral organoids recapitulate some aspects of neural network connectivity, these assays may provide important insights into the neurotoxicity of specific agents on human neurons. The most‐used techniques to evaluate neuronal activity in cerebral organoids are live calcium imaging, MEA, and patch clamping. Patch clamping evaluates the electrophysiological properties of individual cells and requires highly specialized equipment and expertise, which may not be available to many scientists. On the other hand, although calcium imaging and MEA require specific pieces of equipment (fluorescent microscopes and MEA plate reader, respectively), they do not require a high level of technical expertise. Calcium imaging allows investigators to evaluate neuronal activity at the single‐cell level with a temporal resolution of seconds. For each recorded cell, it is possible to determine the change in relative fluorescence over time and the frequency, amplitude, and length of individual calcium influx in each cell. Data obtained are generally presented as individual traces of the relative change in fluorescence in each cell (Fig. 5A) or a raster plot showing individual calcium events for every cell over time (Fig. 5B; see Data Availability Statement for a link to the Python scripts). A calcium event is recorded when a significant change in calcium‐associated fluorescence is observed (typically an increase in fluorescence of 30% to 100% compared with baseline). We and others have developed scripts to extract and graph the relative change in fluorescence over time, and we direct the reader to the following references for more information about how to construct these plots: Giovannucci et al. (2019), Parmentier et al. (2022), Pnevmatikakis (2019), Romano et al. (2017).

**Figure 6:**
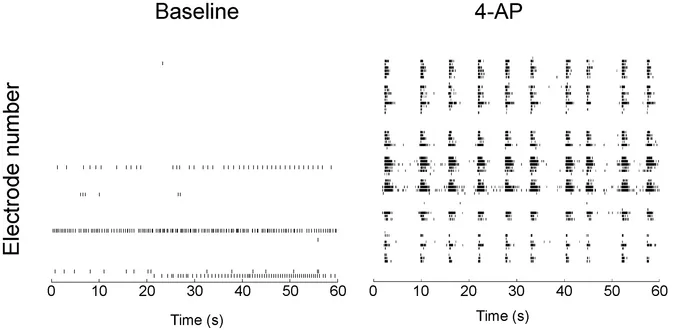
Effect of 4‐aminopyridine (4‐AP) on neuronal activity in cerebral organoids measured with microelectrode arrays. Raster plots of neuronal activity before (left) and after (right) incubation with 4‐AP. Note the appearance of synchronized activity after exposure to 4‐AP. Recording of an organoid at day 160.

Common statistical tests can then be applied to compare the frequency, amplitude, and length of calcium events between control and treated organoids. Alternatively, using the discriminability index *d'* in signal detection may offer more reliable detection of neuronal spikes inferred from changes in calcium fluorescence (Wilt et al., 2013). Because fluorescent calcium indicators are progressively washed out from the cells and can be toxic, calcium imaging cannot be repeated in the same organoids across several days. If this is needed, adding a genetically encoded calcium indicator may be a good alternative approach (Lin & Schnitzer, 2016). To complement calcium imaging, MEA recordings are an attractive technique owing to their high temporal resolution. The microelectrodes do not record the activity of individual neurons but of different regions of the organoid that are in contact with the microelectrodes. This allows evaluation of the synchronization of these different regions. The electrical potential of each electrode can be filtered and analyzed using software such as MATLAB (MathWorks) or Axion Neural Metric Tool (Axion Biosystems), among others. Common statistical tests are used to evaluate spike frequency, burst frequency (rapid succession of spikes for a given electrode), network bursts (electrode bursts happening simultaneously in several electrodes), and synchronization. Data are typically displayed as a raster plot showing the time stamps of spikes for each electrode (Fig. 6). This allows direct visual comparison of the effect of a compound on neural activity in cerebral organoids. Moreover, MEA recording can be performed successively in developing cerebral organoids and may be used to assess the correct development of organoids by evaluating the appearance of spontaneous neuronal activity that is expected to occur.

**Video S1:** Example of a typical calcium imaging recording in cerebral organoids; the frame rate was increased to 13 frames per second with a recording rate of 2 Hz.

### Time Considerations

Cerebral organoid culture can be a lengthy process, especially if mature organoids obtained at later time points are required. Before 30 days of culture, organoids are primarily composed of actively dividing neural precursor cells. Postmitotic neurons (MAP2‐positive, NeuN‐positive neurons) appear around 30 days. Neurons progressively acquire mature electrophysiological features with time in culture. Mature neurons are commonly observed after 120 days in culture. Astrocytes appear between 60 and 90 days in culture, and their proportion increases steadily with time in culture (Parmentier et al., 2022; Paşca et al., 2015; Trujillo et al., 2019). A postnatal gene expression signature was noted in cerebral organoids around 250 days (Gordon et al., 2021). Generating embryoid bodies (Basic Protocol 1 steps 1 to 8) should take ∼2 hr. Medium changes for cerebral organoids will take 30 to 60 min depending on the number of plates. With experience, users can confidently generate embryoid bodies using up to three 96‐well plates at a time. EdU incorporation (Basic Protocol 2) should take ∼3 hr, whereas organoid processing and EdU staining will take around 4 hr of hands‐on time with two overnight incubation steps. Calcium imaging for cerebral organoids takes around 3 to 5 hr depending on the number of recording sessions. Similarly, MEA recordings should take around 2 to 3 hr depending on the number of plates and recording sessions.

### Author Contributions

**Thomas Parmentier**: conceptualization, data curation, formal analysis, investigation, methodology, project administration, validation, visualization, writing–original draft, writing–review and editing; **Jonathan LaMarre**: funding acquisition, project administration, resources, supervision, writing–review and editing; **Jasmin Lalonde**: funding acquisition, project administration, resources, supervision, writing–original draft, writing–review and editing.

### Conflict of Interest

The authors declare no conflicts of interest.

## Supporting Information

Video S1

*Example of a typical calcium imaging recording in cerebral organoids; the frame rate was increased to 13 frames per second with a recording rate of 2 Hz*.

## Data Availability

The code for calcium imaging is available at https://github.com/Thomas‐Parmentier/calcium‐imaging‐cultured‐cells.
